# (−)-Epicatechin—An Important Contributor to the Antioxidant Activity of Japanese Knotweed Rhizome Bark Extract as Determined by Antioxidant Activity-Guided Fractionation

**DOI:** 10.3390/antiox10010133

**Published:** 2021-01-18

**Authors:** Urška Jug, Katerina Naumoska, Irena Vovk

**Affiliations:** 1 Department of Food Chemistry, National Institute of Chemistry, Hajdrihova 19, SI-1001 Ljubljana, Slovenia; urska.jug@ki.si; 2 Faculty of Chemistry and Chemical Technology, University of Ljubljana, Večna pot 113, SI-1000 Ljubljana, Slovenia

**Keywords:** *Polygonum cuspidatum*, Reynoutria, invasive species, phenolic compounds, flavan-3-ols, stilbenes, vitamin C, size-exclusion chromatography, DPPH test, DPPH derivatization

## Abstract

The antioxidant activities of Japanese knotweed rhizome bark extracts, prepared with eight different solvents or solvent mixtures (water, methanol, 80% methanol_(aq)_, acetone, 70% acetone_(aq)_, ethanol, 70% ethanol_(aq)_, and 90% ethyl acetate_(aq)_), were determined using a 2,2-diphenyl-1-picrylhydrazyl (DPPH) free radical-scavenging assay. Low half maximal inhibitory concentration (IC_50_) values (2.632–3.720 µg mL^−1^) for all the extracts were in the range of the IC_50_ value of the known antioxidant ascorbic acid at t_0_ (3.115 µg mL^−1^). Due to the highest extraction yield (~44%), 70% ethanol_(aq)_ was selected for the preparation of the extract for further investigations. The IC_50_ value calculated for its antioxidant activity remained stable for at least 14 days, while the IC_50_ of ascorbic acid increased over time. The stability study showed that the container material was of great importance for the light-protected storage of the ascorbic acid_(aq)_ solution in a refrigerator. Size exclusion–high-performance liquid chromatography (SEC-HPLC)–UV and reversed phase (RP)-HPLC-UV coupled with multistage mass spectrometry (MS^n^) were developed for fractionation of the 70% ethanol_(aq)_ extract and for further compound identification, respectively. In the most potent antioxidant SEC fraction, determined using an on-line post-column SEC-HPLC-DPPH assay, epicatechin, resveratrol malonyl hexoside, and its in-source fragments (resveratrol and resveratrol acetyl hexoside) were tentatively identified by RP-HPLC-MS^n^. Moreover, epicatechin was additionally confirmed by two orthogonal methods, SEC-HPLC-UV and high-performance thin-layer chromatography (HPTLC) coupled with densitometry. Finally, the latter technique enabled the identification of (−)-epicatechin. (−)-Epicatechin demonstrated potent and stable time-dependent antioxidant activity (IC_50_ value ~1.5 µg mL^−1^) for at least 14 days.

## 1. Introduction

Japanese knotweed (*Fallopia japonica* Houtt.; synonyms: *Polygonum cuspidatum* Siebold & Zucc., *Reynoutria japonica* Houtt., *Polygonum reynoutria* Houtt., *Pleuropterus cuspidatus* (Siebold and Zucc.) H. Gross, *Tiniaria japonica* (Houtt.) Hedberg), which is native to East Asia, is an invasive plant species in Europe and North America [1]. The Japanese knotweed rhizome has already been tested in various biological studies [2], and its extract or existing compounds showed antioxidant [3,4,5,6,7,8,9,10,11], estrogenic [12], antiproliferative [3], antibacterial [13], antiviral (anti-human immunodeficiency virus) [14], anti-inflammatory [15], antiatherosclerotic [16] activities, etc. A lot of health benefits of Japanese knotweed rhizome extract were correlated with the content of some antioxidant compounds [4].

Mechanisms of antioxidant activity, such as free radical scavenging, singlet oxygen quenching, transition metal chelation, enzyme mimetic activity, and enzyme inhibition, have been described [17]. There are several methods for evaluating antioxidant activity [18,19] that are based on different mechanisms and can give results that are not comparable. A universal test does not exist; therefore, the use of at least two different methods is strongly recommended [18]. The methods can be classified according to their performance (“in vitro” and “in vivo”) [19], the type of the measurement (e.g., spectrophotometric [3,5,6,7,8,9,10,11], electrochemical [20,21], chromatographic (gas chromatography [22], HPLC [20,21], HPTLC [23,24,25,26])), and the type of the reaction used for the assay (hydrogen atom transfer (HAT)-based assays and electron transfer (ET)-based assays) [18,21].

Among the free radical-scavenging methods, the DPPH assay is the fastest, the most straightforward, relatively inexpensive, efficient, and, therefore, the most frequently employed. Many studies on DPPH assay-guided fractionation of various plant materials have already been performed [27,28,29,30,31,32,33,34,35,36,37,38,39,40], including *on-line* methods with pre-column [27] or post-column [28,29,30,31,32,33,41] DPPH reactions. *On-line* methods for measurement of the free radical-scavenging activity indicate the antioxidant fractions/compounds in a fast and inexpensive way, without the need to isolate and test them *off-line*, which is time consuming, as described in [28].

The antioxidant activity of the Japanese knotweed rhizome has been tested and confirmed using various assays: DPPH radical-scavenging capacity [3,4,5,7,8,9,10,11]; superoxide-scavenging (nitroblue tetrazolium (NBT) reduction) capacity [4]; 2′-azinobis-[3-ethylbenzthiazolin-6-sulfonic acid] (ABTS) radical-scavenging capacity [5,6]; electron spin resonance spectrometry (ESR) [3]; oxygen radical absorption capacity (ORAC) [6]; ferric-reducing antioxidant power (FRAP) [9]; chemiluminescence [5]; phosphomolybdenum reduction [10]; lipid peroxidation inhibition performed on linoleic acid [10] and on mouse brain tissue [4]; DNA strand scission assay [3,4]; and superoxide dismutase (SOD) inhibition assay–water-soluble tetrazolium salt-1 (WST-1) [8].

Tests for determining the total polyphenol content, such as the Folin–Ciocalteu assay [4,5,9,10] have also been frequently used to estimate the antioxidant capacity of Japanese knotweed rhizomes, as the polyphenol content is generally significantly correlated to the sample’s total antioxidant activity [9,18,42,43]. Phenolic compounds act as reducing agents, hydrogen donors, singlet oxygen quenchers, and metal chelators [44].

The antioxidant activities of the extracts obtained from the rhizome of Japanese knotweed and from two other knotweed species, giant knotweed (*Fallopia sachalinensis* Schm.) and their hybrid Bohemian knotweed (*Fallopia×bohemica* Chrtek & Chrtková), using different solvents, have already been compared [10]. The choice of solvent was shown to be of great importance for the extraction of antioxidants [10]. The relationship between the antioxidant activity and the chemical content was determined using principal component analysis (PCA) [10], showing that proanthocyanidins are the most important contributors to the total antioxidant capacity [10].

Japanese knotweed rhizome extract is already commercially available as food supplements, marketed as a source of resveratrol as an antioxidant from the stilbenes. Analyses of the bioactive compounds of Japanese knotweed rhizome extract were predominantly performed by (ultra)high-performance liquid chromatography coupled with a UV detector and (multistage) mass spectrometry ((U)HPLC-UV-MS^(n)^) [6,8,10,45,46,47,48,49,50,51] using RP stationary phase, although HPLC-UV [11,12], HPTLC [52,53,54,55], HPTLC-MS^n^ [52,53], and capillary electrophoresis [56,57] were also used.

The objectives of our work were: (1) to select the most suitable solvent or solvent mixture for the extraction of antioxidants from Japanese knotweed rhizome bark; (2) to determine the antioxidant activity of Japanese knotweed rhizome bark extract; (3) to determine the stability of the antioxidant activity of the selected extract over time; (4) to fractionate the extract by a new SEC-HPLC method and to determine its most potent antioxidant fraction by an *on-line* post-column reaction with DPPH; and (5) to further identify the compounds present in the isolated antioxidant SEC fraction(s) by RP-HPLC-MS and HPTLC.

## 2. Materials and Methods

### 2.1. Chemicals and Materials

All solvents were at least of analytical grade. Methanol (HPLC and LC-MS grade), acetone, and acetonitrile (LC-MS grade) were obtained from Honeywell Reagents (Seelze, Germany). Ethanol (absolute anhydrous) was purchased from Carlo Erba Reagents (Val de Reuil, France). Ethyl acetate, acetic acid (glacial (100%) and glacial (100%) LC-MS grade), concentrated hydrochloric acid (37%), and 4-(dimethylamino)cinnamaldehyde (DMACA) were acquired from Merck (Darmstadt, Germany). Ammonium acetate, 2,2-diphenyl-1-picrylhydrazyl (DPPH), (−)-epicatechin (90%), and (−)-catechin (98%) were acquired from Sigma-Aldrich (Steinheim, Germany). Ascorbic acid was obtained from Fluka, Sigma-Aldrich (Steinheim, Germany). (−)-Epicatechin (of high purity) was purchased from Fluka Chemie (Buchs, Switzerland), while (+)-catechin (98%) was obtained from Carl Roth (Karlsruhe, Germany). A Milli-Q water purification system (18 MΩ cm^−1^; Millipore, Bedford, MA, USA) was used to obtain ultrapure water. Disposable plastic cuvettes were purchased from Brand (Wertheim, Germany).

### 2.2. The Preparation, Extraction Yield, and Antioxidant Activity of Various Extracts

Japanese knotweed (*Fallopia japonica* Houtt.) rhizomes were harvested in Ljubljana, Slovenia (Vrhovci, by a bridge over the Mali Graben, N 46°02′33.9″; E 14°27′00.9″). A voucher specimen was deposited in the Herbarium LJU (LJU10143477). After the rhizomes were cleaned with tap water, the bark was peeled and lyophilized at −50 °C for 24 h (Micro Modulyo, IMAEdwards, Bologna, Italy). The obtained dry material was frozen using liquid N_2_ and pulverized by a Mikro-Dismembrator S (Sartorius, Göttingen, Germany) for 1 min at a frequency of 1700 min^−1^. The lyophilized and pulverized rhizome bark (200 mg; eight replicates) was extracted with 2 mL of the following solvents or solvent mixtures: water, methanol, 80% methanol_(aq)_, acetone, 70% acetone_(aq)_, ethanol, 70% ethanol_(aq)_, and 90% ethyl acetate_(aq)_, followed by 5 min vortexing, 15 min ultrasonication, and 5 min centrifugation at 6700× *g*.

The supernatants were transferred into pre-weighted glass storage vials, where the solvents were evaporated under N_2_ flow. The vials with obtained dry extracts of Japanese knotweed rhizome bark (JKRB) were weighed to calculate the extraction yield. The dry extracts were further dissolved in methanol (stock solutions, which also served as first working solutions: 400 µg mL^−1^) and diluted with the same solvent to obtain additional working solutions with the following concentrations (µg mL^−1^): 200, 100, 50, 25, 12.5, 6.25, 3.125, 1.563, 0.781, 0.391, and 0.195. Immediately after dilution, they were tested using the DPPH assay described in [58]. The DPPH reagent (1 mL of 200 µM methanolic solution of DPPH) was added to 3 mL of each working solution in triplicate (solution A) [59].

To prepare the sample blanks, 1 mL of methanol was added to 3 mL of separate working solutions (solution B) [59]. A control sample (for DPPH) was prepared by the addition of 1 mL of DPPH reagent to 3 mL of methanol in triplicate (solution **C**) [59]. All prepared solutions were vortexed for 5 s and stored in amber glass storage vials for 30 min in the dark at room temperature. Spectrophotometric measurements of the absorbances of solutions A, B, and C (named A_A_, A_B_, and A_C_, respectively; Equation (1)) were performed at 517 nm using a Lambda 45 UV/Vis spectrometer (Perkin Elmer, Waltham, MA, USA) with methanol as a blank solvent for the instrument. The IC_50_ values were calculated and the curves were plotted in GraphPad Prism 7 [60].

Calculation of the DPPH scavenging effect [59]:

DPPH scavenging effect (%) = 100 − ((A_A_ − A_B_) × 100/A_C_) (1)

in which A_B_ is included in the case of yellow-colored working solutions to exclude their absorbance contributions [59].

### 2.3. Comparison between the Antioxidant Activities of the 70% Ethanol_(aq)_ Extract of Japanese Knotweed Rhizome Bark and Ascorbic Acid over Time

A DPPH assay of the selected dry 70% ethanol_(aq)_ extract, re-dissolved in methanol (400 µg mL^−1^) and diluted to the concentrations (µg mL^−1^): 200, 100, 50, 25, 12.5, 6.25, 3.125, 1.563, 0.781, 0.391, and 0.195, and a DPPH assay of ascorbic acid dissolved in methanol (1000 µM) and diluted to the concentrations (µM): 500, 250, 100, 50, 40, 30, 20, 10, and 1, were performed at t = 0, 2, 4, 6, 8, 10, 24, and 50 h, 7 and 14 days (at T = 25 °C) after the preparation of solutions.

The influence of glass vs. plastic storage containers on the stability of ascorbic acid was studied by a 24 h aging of 50 µM aqueous ascorbic acid solutions stored in the refrigerator (T = 4 °C) or at room temperature (T = 25 °C) and: (i) protected from light in plastic centrifuge tubes (T = 4 °C), (ii) protected from light in glass storage vials (T = 4 °C), (iii) exposed to daylight in plastic centrifuge tubes (T = 25 °C), and (iv) exposed to daylight in glass flasks (T = 25 °C). HPLC-UV analyses of ascorbic acid solutions were performed at t = 0 h and at t = 24 h after solution preparation using an in-house HPLC method (confidential) at 254 nm. As ascorbic acid degrades very quickly, three fresh ascorbic acid solutions were prepared at t = 24 h to confirm the intermediate precision of the method (*n* = 6; *t*_R_ = 3.1 min).

### 2.4. SEC-HPLC-UV Fractionation of the 70% Ethanol_(aq)_ JKRB Extract Guided by an On-Line Post-Column Reaction with DPPH

The SEC-HPLC-UV method was developed for the fractionation of the 70% ethanol_(aq)_ extract of JKRB using an Agilent Bio SEC-3 column (150 mm × 4.6; 3 µm, 100 Å) on an HPLC-PDA Agilent Technologies 1260 Infinity system (Santa Clara, California, USA), equipped with a fraction collector (Agilent 1260 Infinity II). OpenLAB CDS ChemStation software (Agilent) was used for data collection and analysis. A pre-mixed mobile phase was prepared with 150 mM ammonium acetate buffer and ethanol in the ratio 75:25 (*v/v*).

The ammonium acetate buffer was prepared by dissolving 5.778 g of ammonium acetate in 500 mL ultrapure water, and acetic acid was used to adjust the pH value to 4.8. An isocratic elution was performed with a flow rate of 0.325 mL min^−1^ and a run time of 40 min. The temperatures of the column and autosampler were set to 40 °C and 25 °C, respectively. The dry 70% ethanol_(aq)_ extract of JKRB was re-dissolved in the mobile phase to achieve a concentration of 0.5 mg mL^−1^ and was filtered through a 0.45 µm polyvinylidene fluoride (PVDF) membrane filter before injection (5 µL). Chromatograms were recorded at different wavelengths (280, 300, and 360 nm), and absorption spectra were acquired as well.

To determine the antioxidant fractions, an *on-line* post-column reaction was performed using DPPH solution (400 µM in 80% methanol_(aq)_) delivered at a flow rate of 5 µL min^−1^ through a syringe pump, leading to one inlet of a T-unit. The second inlet of the T-unit was connected to the column effluent capillary, while the outlet led to a 3.5 m long reaction coil (0.13 mm internal diameter (I.D.)) and later to the photodiode array (PDA) detector. The chromatographic conditions were as explained above. The reaction coil allowed a longer contact time between the eluting fractions’ compounds and the DPPH reagent, thus enabling radical scavenging reactions before reaching the PDA detector. UV/Vis spectra were acquired, and the chromatograms were recorded at 280 and 517 nm.

The decrease in absorbance at 517 nm indicated the antioxidant activity of the fractions, visible as negative peaks on the chromatogram. The *on-line* post-column reaction of the SEC fractions was performed in triplicate. Blank and control analyses were executed as follows: (i) injection of the sample extract and post-column introduction of 80% methanol_(aq)_; (ii) injection of the procedural blank (the mobile phase filtered through a 0.45 µm PVDF membrane filter) and post-column reaction with DPPH; and (iii) injection of the procedural blank and post-column introduction of 80% methanol_(aq)_.

As expected, the reaction coil led to a shift of the retention times (*t*_R_s) to higher values. Therefore, it was used for all analyses, including fraction collection, although the reaction with DPPH was not applied during this step.

Fourteen fractions, detected at 280 nm, were selected for retention time-based collection (Section 3.4). The temperature of the fraction collector was maintained at 4 °C. The collected fractions were pooled, the solvent was evaporated under N_2_ flow, and the solid residues were stored in a freezer at −20 °C.

### 2.5. Analyses of SEC Fractions and Determination of the Strongest Antioxidant by RP-HPLC-MS

The compounds of the isolated SEC fractions were analyzed using a UHPLC-UV-MS system (Accela 1250, coupled to an LTQ Velos MS, Thermo Fisher Scientific, Waltham, MA, USA). A new HPLC-UV-MS method was developed for the separation and characterization of the compounds from the 70% ethanol_(aq)_ extract of JKRB using a Hypersil ODS column (150 × 4.6 mm; 5 µm I.D., Thermo A). SEC fractions (FRs) obtained from 122 (FRs 1–7, 9, and 14), 96 (FRs 10–13), and 77 (FR 8) runs were dried under a N_2_ flow, dissolved in 150 (FRs 2, 5, and 7), 200 (FRs 1, 3, and 4), 250 (FRs 6 and 9), 300 (FR 8), 500 (FR 14), and 1000 µL (FRs 10–13) of solvent (water:ethanol, 3:1, *v/v*) and injected in different volumes (5 µL: FRs 10–13; 10 µL: FRs 1, 6, 9, and 14; 15 µL: FRs 2–5, 7, and 8).

These values were adapted to the peak heights and widths of the SEC fractions. The mobile phase, consisting of 0.1% acetic acid_(aq)_ (A) and acetonitrile (B), and a linear gradient elution with a flow rate of 0.7 mL min^−1^ of 10–100% B (0–30 min), were used. The column and autosampler temperatures were maintained at 25 °C and 10 °C, respectively. Chromatograms were recorded at 280, 300, and 360 nm, and absorption spectra were acquired as well. For the ionization of compounds, heated electrospray ionization (HESI) in negative ion mode was used, and the MS parameters were as follows: heater and capillary temperatures of 400 and 350 °C, respectively, sheath gas 30 arbitrary units (a.u.), auxiliary gas 5 a.u., sweep gas 0 a.u., spray voltage 4 kV, and S-Lens RF level 69.0%.

To optimize the MS parameters, a methanolic standard solution of (−)-epicatechin (0.1 mg mL^−1^, 10 μL min^−1^) was combined with the column effluent (55% B, 0.7 mL min^−1^) using a T-unit, thus directing the combined flow into the MS source. The MS spectra were recorded in the *m/z* range of 50–2000, while the precursor ions of interest were fragmented in MS^n^ using a collision energy of 35%. Xcalibur software (version 2.1.0, Thermo Fisher Scientific) was used to evaluate the collected chromatograms and spectra.

### 2.6. Identification of the Compounds in the Antioxidant Fraction by Orthogonal Methods and Confirmation of Their Antioxidant Activity by DPPH Assay

To confirm the presence of (−)-epicatechin in the isolated antioxidant fraction, commercially available standards of flavan-3-ols were used. Standards of (+)-catechin and (−)-epicatechin were used for the SEC-HPLC-UV and RP-HPLC-UV-MS analyses, while for the HPTLC analysis performed on an HPTLC cellulose stationary phase, which enables separation of enantiomers, a (−)-catechin standard was also applied. Standards were prepared in concentrations of 0.01 mg mL^−1^ (in water:ethanol (3:1, *v/v*) for SEC-HPLC-UV) and 0.1 mg mL^−1^ (in methanol for RP-HPLC-UV-MS and HPTLC). The injection volume was 5 µL for both HPLC methods.

The antioxidant fraction FR 8, collected from 77 runs (by SEC-HPLC method; Section 2.4), was dissolved in 200 µL of methanol. All standards (4 µL, 0.4 µg) and the antioxidant fraction (40 µL) were applied on an HPTLC cellulose plate (Merck, Art. No. 1.05786.0001, cut to 10 cm × 10 cm) as 8 mm bands, 8 mm from the bottom of the plate using a Linomat 5 (Camag, Muttenz, Switzerland). The plate was developed up to 90 mm (45 min) in a normal developing chamber (for 10 cm × 10 cm plates, Camag) using water as a developing solvent [23,61,62] and dried with a stream of warm air for 3 min after development.

Post-chromatographic derivatization was performed by immersing the plate for 1 s into DMACA detection reagent, prepared by dissolving 60 mg of DMACA in 13 mL of concentrated hydrochloric acid (37%) and diluted with ethanol to make up a total volume of 200 mL [61]. The plate was then dried with warm air for 2 min. The DigiStore 2 documentation system in conjunction with Reprostar 3 (Camag) was used for the documentation of the chromatograms at 254 nm, 366 nm, and white light illumination after development and 10 min after post-chromatographic derivatization with DMACA reagent.

After derivatization, the plate was also scanned with a slit-scanning densitometer (TLC Scanner 3, Camag) set in absorption/reflectance mode at 655 nm. The selected wavelength was derived from our previously published studies [61,63]. The other settings were as follows: slit length 6 mm, slit width 0.30 mm, and scanning speed 20 mm s^−1^. Both instruments were controlled using winCATS software (version 1.4.9.2001).

As in Section 2.2 and Section 2.3, the spectrophotometric DPPH assay of methanolic solutions of the (−)-epicatechin standard (Sigma-Aldrich; 1000, 500, 250, 100, 50, 40, 30, 20, 10, 1, and 0.1 µM) was performed at t = 0, 2, 4, 6, 8, 10, 24, and 50 h, 7 and 14 days (storage at T = 25 °C) after solution preparation to determine its IC_50_ value for the radical scavenging activity, as well as the stability of its antioxidant activity.

## 3. Results and Discussion

### 3.1. Extraction Yields and Antioxidant Activity of Various Extracts

The extraction of JKRB was performed with water, polar organic solvents (methanol, acetone, and ethanol), and aqueous solutions thereof (80% methanol_(aq)_, 70% acetone_(aq)_, 70% ethanol_(aq)_, and 90% ethyl acetate_(aq)_). The highest extraction yield was achieved by 70% ethanol_(aq)_, and a slightly lower yield was achieved by 70% acetone_(aq)_ (Table 1). Significantly lower extraction yields were obtained with pure ethanol and acetone. The difference between the extraction yields obtained with methanol and 80% methanol_(aq)_ was not significant. Water gave a higher extraction yield than did pure acetone. The lowest extraction yield was obtained using 90% ethyl acetate_(aq)_ (Table 1).

The antioxidant activities of all dry extracts, re-dissolved in methanol, were tested using a DPPH assay. Re-dissolving all dry extracts in the same solvent (methanol) was preferred (providing equal polarity and pH of the reaction medium for all extracts) to enable comparison of the obtained DPPH assay results, as discussed in [58]. As all dry extracts and DPPH were soluble in methanol, this was selected as a reaction medium.

The obtained results of the DPPH radical scavenging assay are expressed as IC_50_ values, which represent the concentration of the antioxidant required to scavenge 50% of the DPPH free radicals and consequently lead to a 50% decrease in the DPPH absorption [64,65,66].

**Figure 1:**
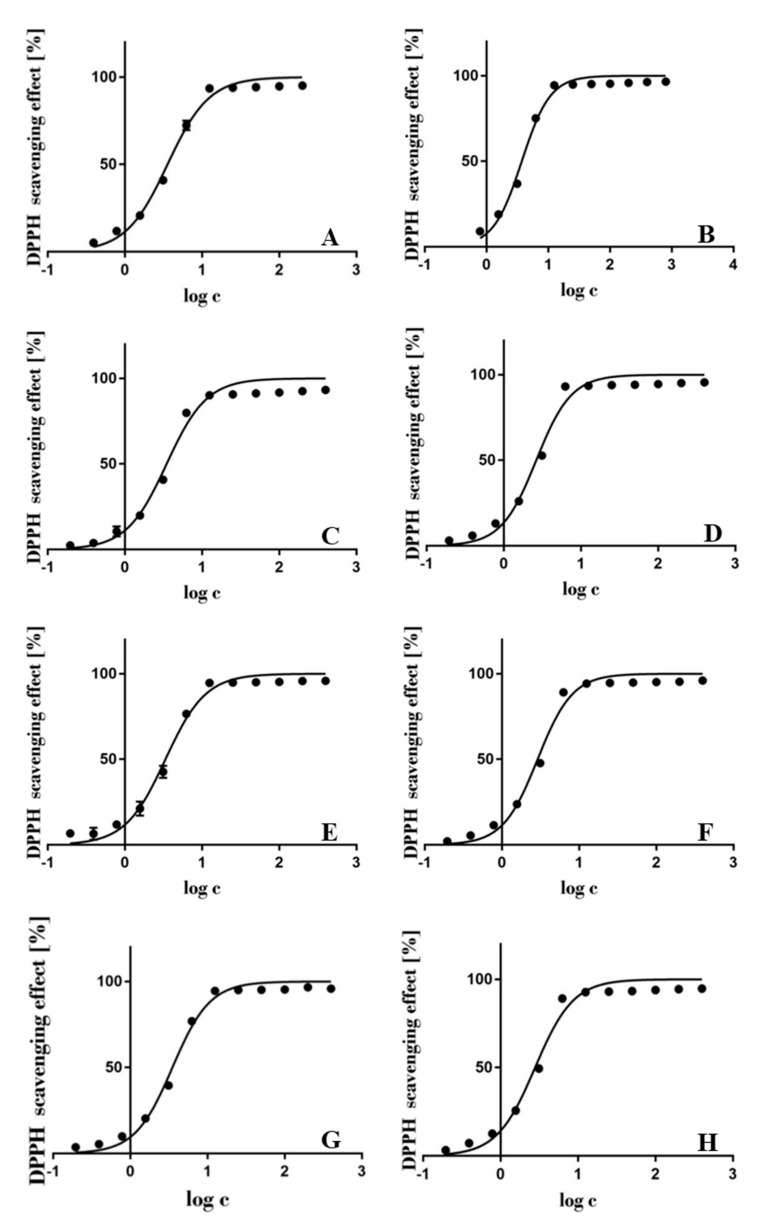
Logarithmic curves of the antioxidant activities of extracts of Japanese knotweed rhizome bark (JKRB) (*n* = 3) prepared with the following solvents and solvent mixtures: water (**A**), methanol (**B**), 80% methanol_(aq)_ (**C**), acetone (**D**), 70% acetone_(aq)_ (**E**), ethanol (**F**), 70% ethanol_(aq)_ (**G**), and 90% ethyl acetate_(aq)_ (**H**). The calculated values of IC_50_ are 3.561 (**A**), 3.715 (**B**), 3.469 (**C**), 2.632 (**D**), 3.350 (**E**), 2.893 (**F**), 3.503 (**G**), and 2.786 (**H**) µg mL^−1^ (obtained by GraphPad Prism 7 [60]).

As different protocols of the same antioxidant assay may lead to incomparable results, a known antioxidant, ascorbic acid, was used as a reference. The IC_50_ values of all JKRB extracts (Figure 1) prepared by different extraction solvents and solvent mixtures were very low (2.632–3.715 µg mL^−1^; Table 1) and in the range of the IC_50_ value of ascorbic acid at t_0_ (3.115 µg mL^−1^; Table 2). This indicates the high antioxidant potential of JKRB extracts, which may be attributed to the activity of the various phenolic compounds present in JKRB [67]. A JKRB extract prepared with 70% ethanol_(aq)_ was used for further analyses due to the highest extraction yield (only 70% acetone_(aq)_ resulted in a comparable yield) (Table 1). Additional reasons for the selection of this green extraction solvent include that ethanol is considered less harmful than other solvents when present as a residual solvent in pharmaceutical formulations [68], 70% ethanol_(aq)_ is suitable for the preparation of tinctures, and ethanol is commercially available as a “food grade” solvent.

The logarithmic curves representing the radical scavenging activity of the extracts of JKRB with different concentrations are shown in Figure 1.

A time-dependent decrease in the antioxidant activity (increase in the IC_50_ value) of the ascorbic acid solutions was observed, which is most likely a consequence of its oxidation, which can particularly be promoted by light, heat, and heavy metal cations [69]. Therefore, the preparation of the ascorbic acid solutions was carried out very quickly, and the time from their preparation to exposure to DPPH was kept as short as possible (10 min in the worst-case scenario). The addition of the chelating agent ethylenediaminetetraacetic acid (EDTA) to ascorbic acid solution was previously found to have an indirect stabilizing effect on the ascorbic acid molecule through the chelation of traces of heavy metals residing on the surface of glass containers [70]. To examine the influence of glass vs. plastic storage containers on the stability of ascorbic acid in solution, its content after storage in different containers was determined by the use of the HPLC-UV method (Section 3.2).

### 3.2. Antioxidant Activity over Time—Ascorbic Acid Compared to the JKRB 70% Ethanol_(aq)_ Extract

**Figure 2:**
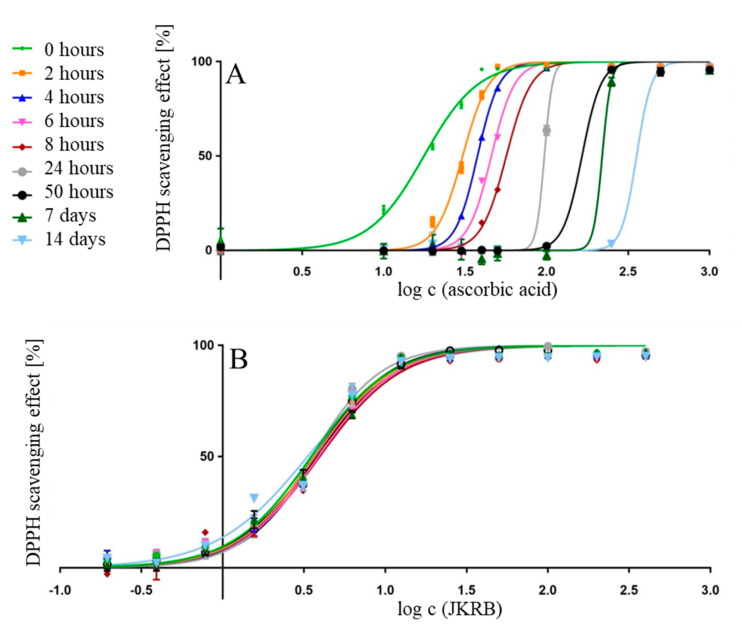
Logarithmic curves plotting the 2,2-diphenyl-1-picrylhydrazyl (DPPH) scavenging effect (%) of ascorbic acid (**A**) and JKRB 70% ethanol_(aq)_ extract (**B**) against the concentration, measured over time.

The antioxidant activity of ascorbic acid continuously decreased over time (IC_50_ value increased from 3.115 up to 62.787 µg mL^−1^, Table 2, Figure 2A), while the antioxidant activity of the JKRB 70% ethanol_(aq)_ extract remained constant during the same time interval (0 h to 14 days) (Table 2, Figure 2B). These results suggest a potential use of the JKRB 70% ethanol_(aq)_ extract as a strong antioxidant material. The potential applications might include the formulation of food supplements (e.g., tincture, powder, and solid dosage forms) or its utilization as a food antioxidant. On the other hand, the stability of ascorbic acid (and its antioxidant effect) in various beverages (bottled and left standing) rich in or enriched with ascorbic acid remains questionable.

As the mobile phase used for the HPLC quantification of ascorbic acid was aqueous based, ascorbic acid for the HPLC analyses was dissolved in water. After 24 h of aging in daylight and at room temperature, practically all ascorbic acid was lost (ascorbic acid <1%), regardless of the container material used for storage. On the other hand, the container material was of great importance for the light-protected storage of the ascorbic acid_(aq)_ solution in the refrigerator. After 24 h of aging (dark, refrigerator) in a plastic container, the content of ascorbic acid_(aq)_ was 65.19% of the initial concentration, while storage in a glass container resulted in a loss (<1% of the initial concentration) comparable to that reported for the room conditions (room temperature and daylight).

**Figure 3:**
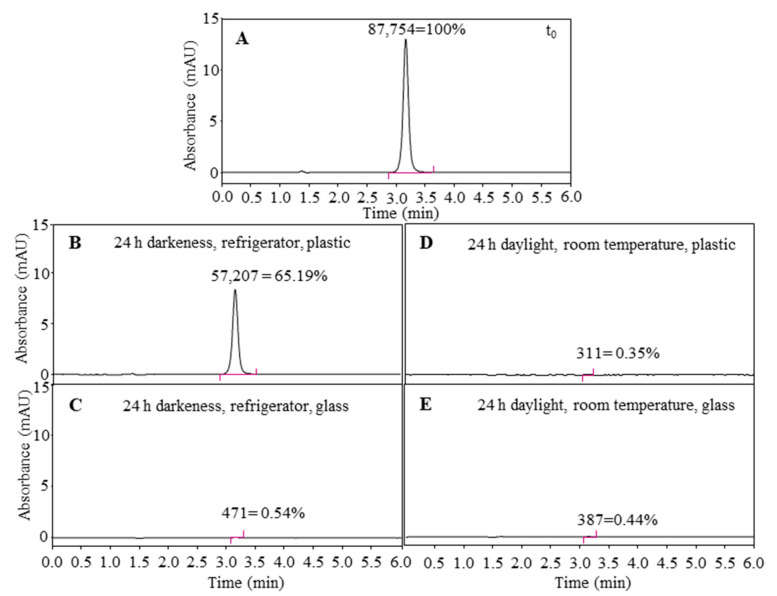
Ascorbic acid_(aq)_ (50 µM) analyzed immediately after preparation (**A**) was subjected to 24 h of aging (**B**–**E**), stored in the refrigerator and protected from light (**B**,**C**) in plastic (**B**) and glass containers (**C**) or stored in daylight at room temperature (**D**,**E**) in plastic (**D**) and glass containers (**E**). The peak areas corresponding to ascorbic acid (*t_R_* 3.1 min, 254 nm) in aged solutions were compared to the peak area of ascorbic acid in the fresh solution. The intermediate precision of the method was 3% (*n* = 6).

Based on these results, the combination of light and temperature, as well as trace metals on the glass surface, influence the stability of ascorbic acid in aqueous solution (Figure 3). On the other hand, the JKRB extract showed stable antioxidant activity for at least 14 days in the worst-case scenario conditions for ascorbic acid (light, room temperature, glass container). The flavan-3-ols, proanthocyanidins, and anthraquinones, which represent major groups of compounds in the JKRB extract [67], are proven chelating agents of glass surface ions [71,72], acting through their hydroxyl or both carbonyl and hydroxyl groups, located on the vicinal or *peri* positions [71]. This supports our findings regarding the stability of the measured antioxidant activity of the JKRB extract.

### 3.3. SEC-HPLC Fractionation of the JKRB 70% Ethanol_(aq)_ Extract, On-Line Post-Column Reaction of the SEC Fractions with DPPH and Determination of the Antioxidant Fractions

A SEC-HPLC-UV method was developed for the first time to separate the compounds from the Japanese knotweed rhizome extract. A SEC column with a pore size of 100 Å was used to enable better separation of the smaller molecules from the extract. A high concentration of the buffer (150 mM) was used to reduce the secondary interactions on the column. Ethanol as a co-solvent, mixed with the buffer in a ratio of 25:75 (*v/v*), improved the solubility of JKRB compounds in the mobile phase. A higher percentage of ethanol in the mobile phase causes precipitation of the ammonium acetate buffer. The chromatograms were recorded at 280 nm, where the highest sensitivity for most of the compounds was achieved. Fourteen of the most abundant fractions (FR 1–FR 14) were selected for isolation.

The antioxidant potential of the Japanese knotweed rhizome bark was tested for the first time using the SEC-HPLC-UV/Vis method with an *on-line* post-column DPPH assay. Finding the right concentration, flow rate, and solvent for the DPPH reagent (insoluble in water and soluble in methanol) to be introduced into the mobile phase (buffer insoluble in methanol) was challenging. However, a 400 µM DPPH solution in 80% methanol_(aq)_, delivered at a flow rate of 5 µL min^−1^, proved to be a good choice as it did not cause precipitations in the system upon contact with the mobile phase. The isocratic elution of the SEC method enabled the constant solubility of the DPPH reagent in the mobile phase and equal chemical reaction conditions throughout the whole run, thus ensuring more relevant results related to the antioxidant activity in comparison to gradient mode chromatography (e.g., RP in the gradient mode).

**Figure 4:**
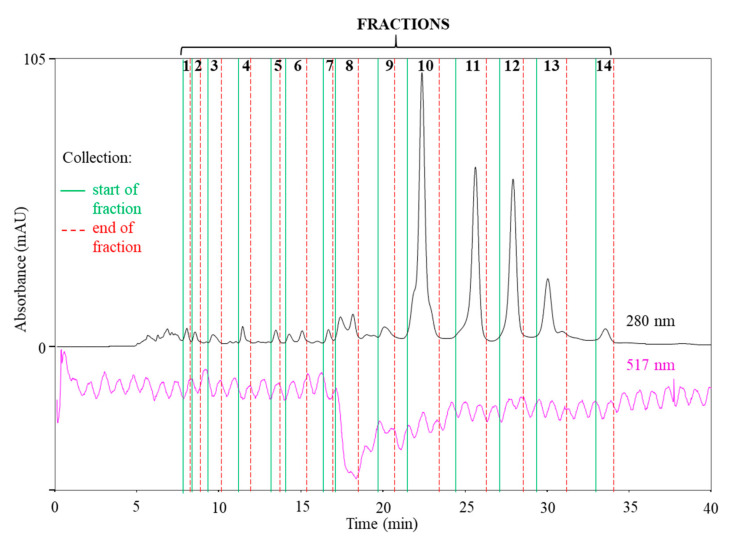
SEC-HPLC-UV/Vis chromatogram at 280 nm (without post-column reaction) and at 517 nm (after post-column reaction with DPPH). The fractions and time intervals selected for fraction collection are marked. Fraction 8 was determined to be the strongest antioxidant due to the decrease in the absorbance at 517 nm.

The noisy baseline of the chromatogram at 517 nm (Figure 4) was most probably due to the imperfections of the in-house built equipment for *on-line* post-column derivatization. Therefore, some antioxidant fractions might have been overlooked, due to a potentially too low decrease in the baseline at 517 nm. However, FR 8, eluting at *t*_R_ 16.8–18 min (Figure 4), was undoubtedly determined as the most potent antioxidant (a clear baseline drop at 517 nm). Although only FR 8 showed antioxidant activity, all fractions (FR 1–FR 14, Figure 4) were collected and screened using RP-HPLC.

### 3.4. Characterization of the Compounds in the Isolated SEC Fractions, Identification of the Antioxidant Fraction Compounds by Orthogonal Methods, and their Antioxidant Activity over Time

An RP-HPLC-MS^n^ method was developed to analyze the compounds in the isolated SEC fractions. The compounds were tentatively identified by comparing the obtained and literature MS and MS^2^ data (Table 3). For the antioxidant fraction FR 8, MS^3^ was also performed. Although expected, the size distribution of the SEC eluting compounds (from larger to smaller molecular masses) was not obvious (Table 3). One of the possible explanations relates to the content of the organic solvent (25%) in the mobile phase, which might promote secondary interactions and might subsequently impact the distribution of the compounds.

**Figure 5:**
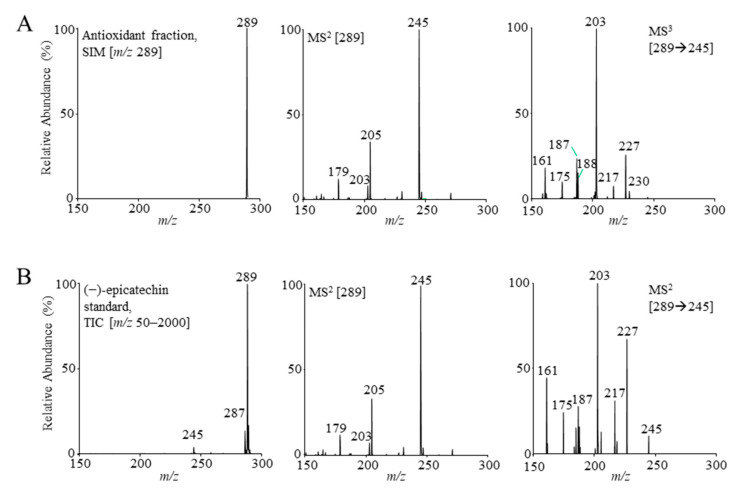
The flavan-3-ol monomer identified by (−)ESI-MS based on the mass spectra and fragmentation patterns obtained for the signal at *t*_R_ 6.4 min in the antioxidant fraction (FR 8) (**A**) and confirmed by (−)-epicatechin standard (**B**). Figure abbreviations: selected ion monitoring (SIM), and total ion current (TIC).

The presence of flavan-3-ol monomer, as the main representative in the antioxidant fraction FR 8, was suspected based on the mass spectra and fragmentation patterns, which were compared to those of the (−)-epicatechin standard (Figure 5) and to the literature data [52,53,67]. MS signals of resveratrol malonyl hexoside, resveratrol, and resveratrol acetyl hexoside were also observed in FR 8, where the last two most likely corresponded to in-source fragments of resveratrol malonyl hexoside [67]. Additional MS signals (Table 3) were not identified due to their low abundance.

**Figure 6:**
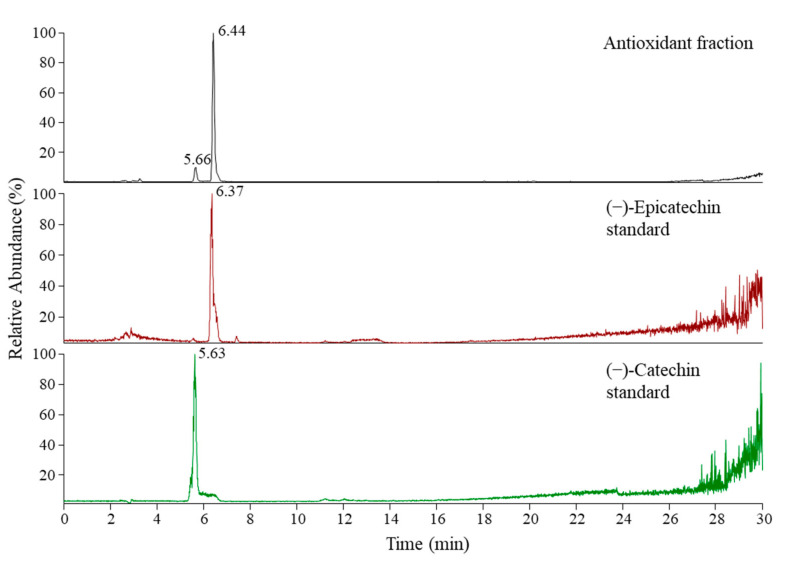
RP-HPLC-MS chromatograms of the antioxidant fraction (FR 8) in SIM mode (*m/z* 289), (−)-epicatechin and (+)-catechin standards (both in TIC mode; *m/z* 50–2000).

In our previous study [67], (+)-catechin and (−)-epicatechin were identified as the two main flavan-3-ol monomers in JKRB. Therefore, both standards were analyzed using the developed RP-HPLC-MS method, which resulted in the separation of (−)-epicatechin (*t*_R_ 6.4 min) and (+)-catechin (*t*_R_ 5.6 min) (Figure 6). The presence of epicatechin (Figure 6) was thus confirmed in FR 8 (Figure 5 and Figure 6).

**Figure 7:**
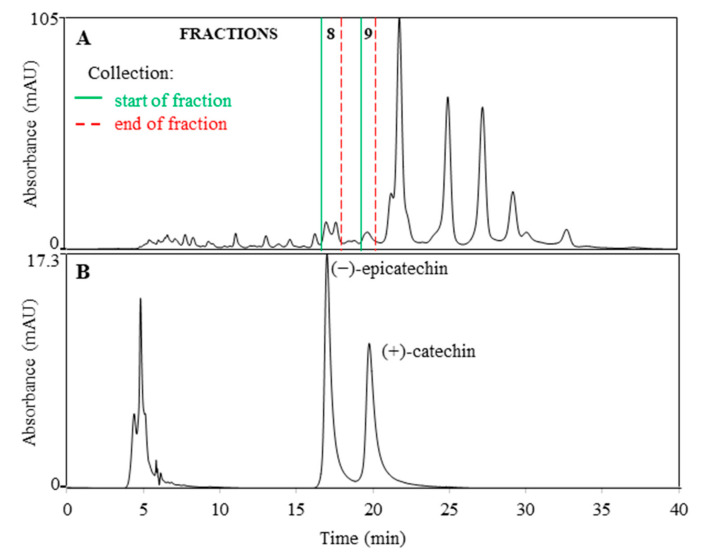
Matching the *t*_R_s of the antioxidant fraction FR 8 (**A**) and (−)-epicatechin (**B**), and the *t*_R_s of FR 9 (**A**) and (+)-catechin (**B**). Chromatograms were acquired at 280 nm using the SEC-HPLC method.

The (−)-epicatechin and (+)-catechin standards were also analyzed using the SEC-HPLC method and the *t*_R_ of (−)-epicatechin (17.2 min) matched the *t*_R_ range of the antioxidant fraction FR 8 (16.8–18.0 min) (Figure 7), while (+)-catechin (*t*_R_ 19.8 min) eluted at the *t*_R_ range of FR 9 (19.4–19.8 min). MS signals of flavan-3-ols at *m/z* 289 were observed by RP-HPLC-MS in both fractions (Table 3). Unexpectedly, catechin and epicatechin diastereoisomers were separated by SEC-HPLC (Figure 7), likely due to their conformational differences or as a consequence of secondary interactions in the column. However, C18-RP-HPLC and SEC-HPLC methods do not enable distinguishing between the enantiomers ((+)-catechin and (−)-catechin; (+)-epicatechin and (−)-epicatechin). According to our previous study [67], the presence of diastereoisomer (−)-epicatechin in FR 8 and (+)-catechin in FR 9, was suspected.

Matching UV spectra of the isolated epicatechin in FR 8 and (−)-epicatechin standard obtained by RP-HPLC and SEC-HPLC methods (λ_max_ at 230 and 280 nm—data not shown) showed that epicatechin is the main compound of the most potent antioxidant fraction, FR 8.

**Figure 8:**
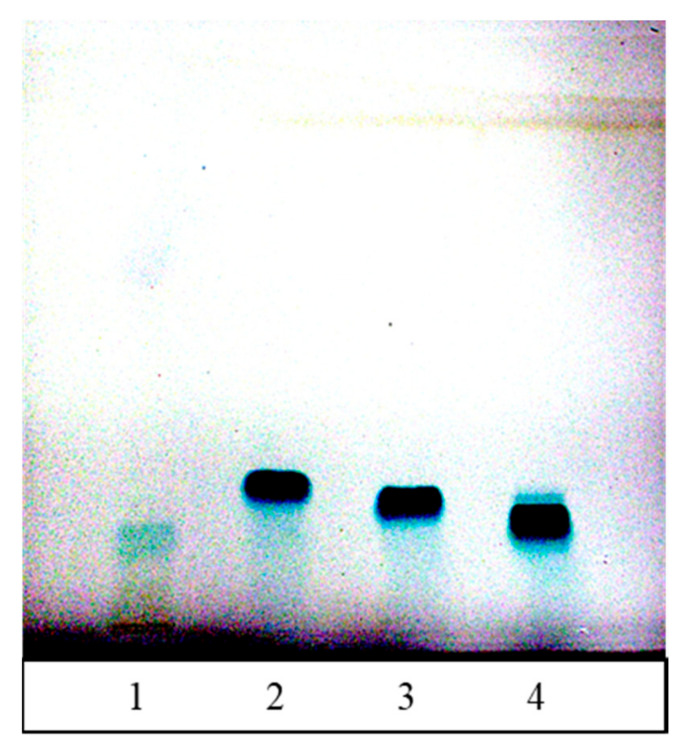
Chromatogram of FR 8 (**track 1**, 40 µL), (+)-catechin (**track 2**, 400 ng), (−)-catechin (**track 3**, 400 ng), and (−)-epicatechin (**track 4**, 400 ng) applied as 8 mm bands on the HPTLC cellulose plate developed with water, derivatized with DMACA reagent, and documented with illumination with white light.

**Figure 9:**
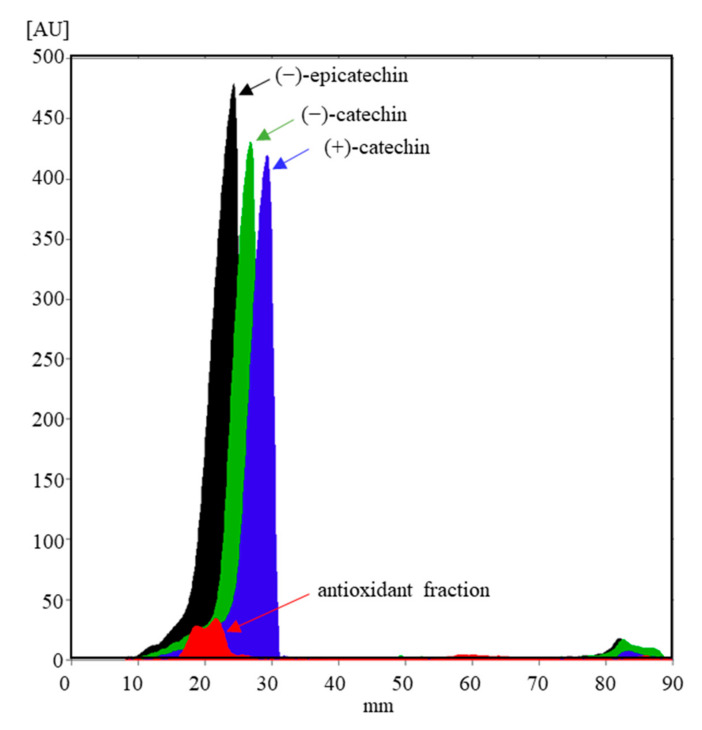
Densitograms of FR 8 (40 µL) and monomeric flavan-3-ol standards (400 ng) scanned at 655 nm in the absorption/reflectance mode on the HPTLC cellulose plate developed with water and derivatized with DMACA.

To confirm the presence of (−)-epicatechin, HPTLC analysis of FR 8 and three standards, (−)-epicatechin, (+)-catechin, and (−)-catechin, was performed on the cellulose stationary phase, which acts as a chiral selector. (+)-Epicatechin was not applied on the plate as it is not commercially available. Derivatization of the chromatograms with the DMACA detection reagent (flavan-3-ol-specific reagent) confirmed the presence of (−)-epicatechin in FR 8 (matching *R*_F_ values of the bands of FR 8 and the (−)-epicatechin standard; Figure 8). In addition to the band for (−)-epicatechin, another poorly resolved band appeared in FR 8 (Figure 8, track 1), which also showed a positive reaction with DMACA. The presence of the two peaks was also confirmed densitometrically (Figure 9).

Resveratrol malonyl hexoside, which was also detected in the antioxidant fraction, was reported for the first time in the Japanese knotweed rhizome in our previous study [67]. Unfortunately, the standard of this compound is not commercially available, thus, an additional confirmation of its presence in the antioxidant fraction was not possible (too low an amount for nuclear magnetic resonance (NMR) spectroscopy).

The antioxidant potential of resveratrol, an aglycone moiety of resveratrol malonyl hexoside, is already well known, and resveratrol’s presence has also been linked to the antioxidant potential of the Japanese knotweed rhizome [8,73]. Resveratrol may cause synergistic or additive antioxidant effects in combination with epicatechin or other extract constituents (Table 3). Recently, an important contribution to the high antioxidant potential of this plant material was attributed to flavan-3-ols and proanthocyanidins [10]. Epicatechin was confirmed in the antioxidant fraction by three orthogonal methods, SEC-HPLC, RP-HPLC-MS, and HPTLC, among which the latter enabled the identification of (−)-epicatechin, which was already recognized as an antioxidant [18,29,42].

We also compared the antioxidant activities of (−)-epicatechin and *trans*-resveratrol standards by DPPH assay. The results showed that (−)-epicatechin is a stronger DPPH radical scavenger than *trans*-resveratrol (higher IC_50_; 7.08 µg/mL or 31.02 µM). Moreover, (−)-epicatechin was shown to be present in higher quantities in Japanese knotweed rhizome bark in comparison to *trans*-resveratrol [11].

**Figure 10:**
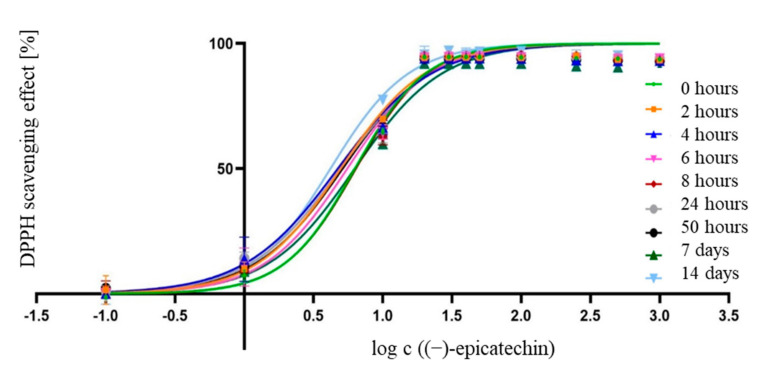
Logarithmic curves plotting the DPPH scavenging effect (%) of (−)-epicatechin against the concentration, measured over time.

As in previous experiments with 70% ethanolic_(aq)_ JKRB extract and ascorbic acid methanolic solutions (Section 3.1 and Section 3.2), a spectrophotometric DPPH assay was performed to determine the IC_50_ value of the free radical scavenging potential of the methanolic solutions of (−)-epicatechin and to test the stability of its antioxidant activity over time (t = 0, 2, 4, 6, 8, 24, and 50 h, and 7 and 14 d). The IC_50_ value of the radical scavenging of (−)-epicatechin was ~1.56 µg mL^−1^, which indicated a higher antioxidant activity compared to that of ascorbic acid. Surprisingly, the antioxidant activity of the (−)-epicatechin standard remained constant over time (0 h to 14 days; ~1.56 µg mL^−1^) (Figure 10, Table 4).

The low IC_50_ value of (−)-epicatechin’s radical scavenging potential and the stability of its antioxidant activity for at least 14 days indicated that (−)-epicatechin could represent one of the most important contributors to the antioxidant activity of the JKRB extract. The reaction of antioxidants with DPPH is influenced by steric hindrance, with a preference for small antioxidant molecules [18]. Therefore, the antioxidant potential of fractions composed of different molecules is only indicative [18]. The results of the radical scavenging activity of JKRB extract, ascorbic acid, and (−)-epicatechin could not be directly compared to the results of other antioxidant assays.

The antioxidant potential of the whole extract may be the result of a synergistic or additive effect of different matrix compounds, which may be even more potent compared to the isolated single compounds’ effect either in the human body [18] or as food antioxidants [76,77]. In the current study, a high time-dependent stability (up to 14 days) of the antioxidant activities of the JKRB extract and (−)-epicatechin (standard solution) was observed.

## 4. Conclusions

Antioxidant activities of Japanese knotweed rhizome bark extracts prepared with water, methanol, 80% methanol_(aq)_, acetone, 70% acetone_(aq)_, ethanol, 70% ethanol_(aq)_, and 90% ethyl acetate_(aq)_ were measured using a DPPH free radical-scavenging assay (IC_50_ = 3.561, 3.715, 3.469, 2.632, 3.350, 2.893, 3.503, and 2.786 µg mL^−1^, respectively). Due to the highest extraction yield, the 70% ethanol_(aq)_ extract was selected for further fractionation.

A SEC method was developed for the first time for fractionation of the Japanese knotweed rhizome (bark) extract. Its antioxidant potential was tested for the first time using the SEC-HPLC-UV/Vis method with an on-line post-column DPPH assay. This approach can also be used for the isolation of other plant bioactive constituents. The compounds in the isolated SEC fractions were determined with a new RP-HPLC-UV-MS^n^ method. Epicatechin was confirmed in the antioxidant fraction by three orthogonal methods, SEC-HPLC-UV, RP-HPLC-MS, and HPTLC, among which the latter enabled the identification of (−)-epicatechin. The antioxidant activity of the (−)-epicatechin standard was additionally proven with a DPPH free radical-scavenging assay.

The IC_50_ values of the antioxidant activity of the selected extract (~3.7 µg mL^−1^) and of (−)-epicatechin (~1.6 µg mL^−1^) remained constant for 14 days, while the IC_50_ values of ascorbic acid increased over time (3.115–62.787 µg mL^−1^). The antioxidant activity of the extract was comparable to that of ascorbic acid at t_0_, while the antioxidant activity of (−)-epicatechin was even higher.

## Figures and Tables

**Table 1:** The extraction yields and the calculated values of the half maximal inhibitory concentrations (IC_50_) of antioxidant activity (GraphPad Prism 7 [60]) of extracts prepared with different solvents or solvent mixtures tested in the concentration range of 0.195–400 µg mL^−1^.

	Extraction Solvents							
	Water	Methanol		Acetone		Ethanol		Ethyl Acetate
		100%	80% _(aq)_	100%	70% _(aq)_	100%	70% _(aq)_	90% _(aq)_
**Extraction yield** (*w/w* %)	25.8	38.1	37.2	21.1	42.6	29.3	44.3	14.9
**IC_50_** (µg mL^−1^)	**3.561**	**3.715**	**3.469**	**2.632**	**3.350**	**2.893**	**3.503**	**2.786**
**LogIC_50_**	0.552	0.570	0.540	0.420	0.525	0.461	0.544	0.445
**LogIC_50_ std. error**	0.016	0.014	0.022	0.018	0.017	0.016	0.014	0.020
**Hillslope**	1.607	1.884	1.669	1.911	1.665	1.924	1.789	1.756
**Hillslope std. error**	0.083	0.105	0.125	0.136	0.097	0.123	0.093	0.124

**Table 2:** The calculated IC_50_ values of the antioxidant activity of ascorbic acid (AA, in the range 1–1000 µM or 0.176–176.12 µg mL^−1^) and JKRB 70% ethanol_(aq)_ extract (in the range 0.195–400 µg mL^−1^) over time.

	0 h	2 h	4 h	6 h	8 h	24 h	50 h	7 d	14 d
**IC_50_ AA** (µM)	**17.6853.1**	**30.524**	**37.662**	**45.846**	**56.612**	**~96.886**	**164.933**	**~219.382**	**356.495**
**IC_50_ AA** (µg mL^−1^)	**15**	**5.376**	**6.633**	**8.075**	**9.971**	**~17.064**	**29.049**	**~38.637**	**62.787**
**LogIC_50_** (µM)	1.248	1.485	1.576	1.661	1.753	~1.986	2.217	~2.341	2.552
**LogIC_50_ std. error**	0.011	0.005	0.003	0.006	0.006	~1.293	0.020	~33.874	0.015
**Hillslope**	2.606	5.344	6.492	6.060	5.673	~17.640	7.444	~16.268	9.400
**Hillslope std. error**	0.149	0.304	0.254	0.566	0.383	~1659.493	0.771	~9712.611	0.938
**IC_50_ JKRB** (µg mL^−1^)	**3.503**	**3.684**	**3.662**	**3.876**	**3.947**	**3.530**	**3.759**	**3.731**	**3.325**
**LogIC_50_**	0.544	0.566	0.564	0.588	0.596	0.548	0.575	0.572	0.522
**LogIC_50_ std. error**	0.014	0.013	0.015	0.017	0.021	0.016	0.011	0.014	0.024
**Hillslope**	1.789	1.815	1.858	1.796	1.736	2.044	1.933	1.737	1.531
**Hillslope std. error**	0.093	0.086	0.106	0.117	0.128	0.140	0.085	0.088	0.118

**Table 3:** RP-HPLC-MS analysis of the compounds in the SEC-HPLC-UV fractions (FR 1–FR 14), corresponding to the SEC *t*_R_ ranges (FR 1: 7.7–8.0, FR 2: 8.3–8.6, FR 3: 9.3–9.8, FR 4: 11.0–11.7, FR 5: 13.0–13.4, FR 6: 13.6–15.0, FR 7: 16.1–16.6, FR 8: 16.8–18.0, FR 9: 19.4–19.8, FR 10: 20.8–22.9, FR 11: 24.0–25.5, FR 12: 26.4–27.8, FR 13: 28.7–30.2, and FR 14: 32.0–32.9). Different numbers of collection runs were performed to isolate the SEC fractions: 122 (FRs 1–7, 9, 14), 96 (FRs 10–13), and 77 (FR 8). Fractions were dissolved in different volumes of water:ethanol (3:1, *v/v*): 150 (FRs 2, 5, 7), 200 (FRs 1, 3, 4), 250 (FRs 6, 9), 300 (FR 8), 500 (FR 14), and 1000 µL (FRs 10–13) and injected in different volumes (5 (FRs 10–13), 10 (FRs 1, 6, 9, 14), and 15 µL (FRs 2–5, 7, 8)) into the RP-HPLC-MS system.

FR	t_R_^a^ [min]	MS [M-H]^−^	MS^2^ and MS^3 b^	Tentatively Identified Compounds
1	6.9	395	[395]: 215	^c^
	10.2, 9.4	1005	[1005]: 713, 917, 961, 458	derivative of emodin bianthrone-hexose-malonic acid [10]
2	7.3	521	[521]: 359	^c^
	7.3	581	[581]: 521, 522, 544, 563, 499, 483, 417	^c^
	7.3	603	[603]: 543, 521	^c^
3	10.2	919	[919]: 713, 671, 875, 458, 509, 416	emodin bianthrone-hexose-(malonic acid)-hexose [10]
	10.2	941	No data	^c^
	11.4, 12.2	933	[933]: 889, 458, 727	methyl derivative of emodin bianthrone-hexose-(malonic acid)-hexose [10]
4	10.2	919	[919]: 713, 671, 875, 458, 416, 509	emodin bianthrone-hexose-(malonic acid)-hexose [10]
	10.2	1005	[1005]: 917, 961, 875, 713, 458	derivative of emodin bianthrone-hexose-malonic acid [10]
	10.2, 12.0	1027	[1027]: 939, 983, 735 (10.2 min)	^c^
			[1027]: 389, 489, 533, 449, 744, 862, 939, 983,	^c^
			994 (12.0 min)	
	12.3	1009	[1009]: 471, 389, 515, 921, 965	^c^
	12.3	987	[987]: 449, 943	^c^
	12.3	449	[449]: 245	torachrysone 8-*O*-(6′-*O*-acetyl)-glucoside [67]
5	6.6, 10.6	473	[473]: 455, 413 (6.6 min)	^c^
			[473]: 269 (10.6 min)	emodin-*O*-(acetyl)-hexoside [67]
	6.6, 10.6	605	[605]: 587	^c^
	6.6, 10.6	665	[665]: 647, 605, 589, 545, 501, 567	^c^
	10.6, 20.6	269	[269]: 225, 269, 251, 241, 187	emodin [67,74]
6	11.2	265	No data	^c^
	11.2	297	No data	^c^
	11.2, 11.8	1005, 502, 458	[1005]: 713, 917, 458	derivative of emodin bianthrone-hexose-malonic acid [10]
	11.2, 11.8	1027	[1027]: 781, 863, 699, 715, 945, 617	^c^
	13.7	1019, 975	[1019]: 691, 773, 855, 609, 527, 937	derivative of bianthrone [10]
	11.2	265	No data	^c^
7	11.6	407	[407]: 245	torachrysone-8-*O*-glucoside/procyanidin degradation product [67]
	13.5	933	[933]: 889, 685, 416	methyl derivative of emodin bianthrone-hexose-(malonic acid)-hexose [10]
	13.5	1019	No data	^c^
8	5.6, 6.3	289	[289]: 245, 205, 179, 203;	(−)-epicatechin (6.32 min) [67], (−)-epicatechin standard
			[289➔245] b: 203, 227, 161, 175, 187, 217, 245	
	9.2, 8.3	431	[431]: 227, 389	resveratrol acetyl hexoside [67]
	9.7, 9.0, 8.3	445	[445]: 385 (9.7 min)	^c^
			[445]: 281, 325, 369, 427, 263, 211 (9.0, 8.3 min)	^c^
	9.2, 8.3	475	[475]: 431	resveratrol malonyl hexoside [67]
	9.2, 8.3	491	[491]: 431	^c^
	9.2, 8.3	227	[227]: 185, 183, 159, 157, 227, 209, 143	resveratrol [67,52]
	5.6, 6.3	289	[289]: 245, 205, 179, 203;	(−)-epicatechin (6.32 min) [67], (−)-epicatechin standard
9	5.6	289	[289]: 245, 205, 203, 179	catechin [67]
	9.9	431	[431]: 269	emodin-*O*-hexoside [67]
	9.9, 20.5	269	[269]: 269, 225, 241, 251, 209, 271	emodin [67,74]
	9.9	385	No data	^c^
10	7.1, 8.9	389	[389]: 227	polydatin (piceid)/resveratroloside [67]
	7.1, 8.9	425d	[425]: 389	polydatin (piceid) (dihydrate)/resveratroloside (dihydrate) [67]
	7.1	449d	[449]: 389, 227	resveratrol acetyl hexoside (hydrate) [67]
	7.1, 8.9	227	[227]: 185, 183, 159, 157, 209, 143, 165	resveratrol [67]
	10.8	473	[473]: 269, 311	emodin-*O*-(acetyl)-hexoside [67]
	10.8	517	[517]: 473, 431	emodin-*O*-(6′-*O*-malonyl)-hexoside [67]
11	6.6, 8.3	405	[405]: 243	piceatannol-3-*O*-glucoside [10]
	6.6, 8.3	243	[243]: 225, 201, 199, 175, 215, 159	piceatannol [75]
12	7.9, 9.1	389	[389]: 227	polydatin (piceid)/resveratroloside [67]
	7.9, 9.1	227	[227]: 185, 183, 209, 159, 157, 165, 143	resveratrol [67]
	7.9, 9.1	425 ^d^	[425]: 389, 227	polydatin (piceid) (dihydrate)/resveratroloside (dihydrate) [67]
13	11.6	431	[431]: 269, 311, 413	emodin-*O*-hexoside [67]
	11.6	269	[269]: 225, 269, 241, 251	emodin [67,74]
	4.9	565	No data	^c^
14	7.2, 9.5	245	[245]: 230 (7.2 min)	^c^ [67]
			[245]: 229 (230) (9.5 min)	^c^ [67]
	7.2, 9.5	325 ^d^	[325]: 245 (7.2 min)	catechin dihydrate/unknown [67]
	7.2, 9.5	245	[325]: 244 (245), 203, 283 (9.5 min)	catechin dihydrate/unknown [67]

^a^*t*_R_ obtained by RP-HPLC-ESI-MS; ^b^ MS^3^; ^c^ Not identified; ^d^ [M-H+(2)H_2_O].

**Table 4:** The calculated IC_50_ values of the antioxidant activity of (−)-epicatechin tested in the range of 0.1–1000 µM or 0.029–290 µg mL^−1^ over time.

	0 h	2 h	4 h	6 h	8 h	24 h	50 h	7 d	14 d
**IC_50_** (µM)	**6.298**	**4.967**	**4.738**	**5.539**	**6.393**	**4.949**	**5.129**	**6.280**	**4.190**
**IC_50_** (µg mL^−1^)	**1.828**	**1.442**	**1.375**	**1.608**	**1.856**	**1.436**	**1.489**	**1.823**	**1.216**
**LogIC_50_**	0.799	0.696	0.676	0.744	0.806	0.695	0.710	0.798	0.622
**LogIC_50_ std. error**	0.039	0.040	0.047	0.045	0.043	0.044	0.041	0.052	0.039
**Hillslope**	1.690	1.427	1.282	1.441	1.669	1.315	1.367	1.375	1.538
**Hillslope std. error**	0.176	0.110	0.106	0.134	0.188	0.107	0.109	0.153	0.112

## Data Availability

The data presented in this study are available in this article.
